# Lions, tigers and kittens too: ACE2 and susceptibility to COVID-19

**DOI:** 10.1093/emph/eoaa021

**Published:** 2020-07-03

**Authors:** Sabateeshan Mathavarajah, Graham Dellaire

**Affiliations:** e1 Department of Pathology, Faculty of Medicine, Dalhousie University, Halifax, Nova Scotia, Canada; e2 Department of Biochemistry and Molecular Biology, Faculty of Medicine, Dalhousie University, Halifax, Nova Scotia, Canada

**Keywords:** coronavirus, COVID-19, SARS-CoV-2, molecular evolution, ACE2, host, pathogen

## Abstract

SARS-CoV-2 (Severe Acute Respiratory Syndrome coronavirus 2) has been reported to infect domesticated animals in a species-specific manner, where cats were susceptible but not dogs. Using the recently published crystal structure of the SARS-CoV-2 spike protein complexed with the human host cell receptor angiotensin converting enzyme 2 (ACE2), we characterized the structure and evolution of ACE2 in several of these species and identify a single interacting amino acid residue conserved between human and *Felidae* ACE2 but not in *Canidae* that correlates with virus susceptibility. Using computational analyses we describe how this site likely affects ACE2 targeting by the virus. Thus, we highlight how evolution-based approaches can be used to form hypotheses and study animal transmission of such viruses in the future.

## BREVIA

**Figure 1.**
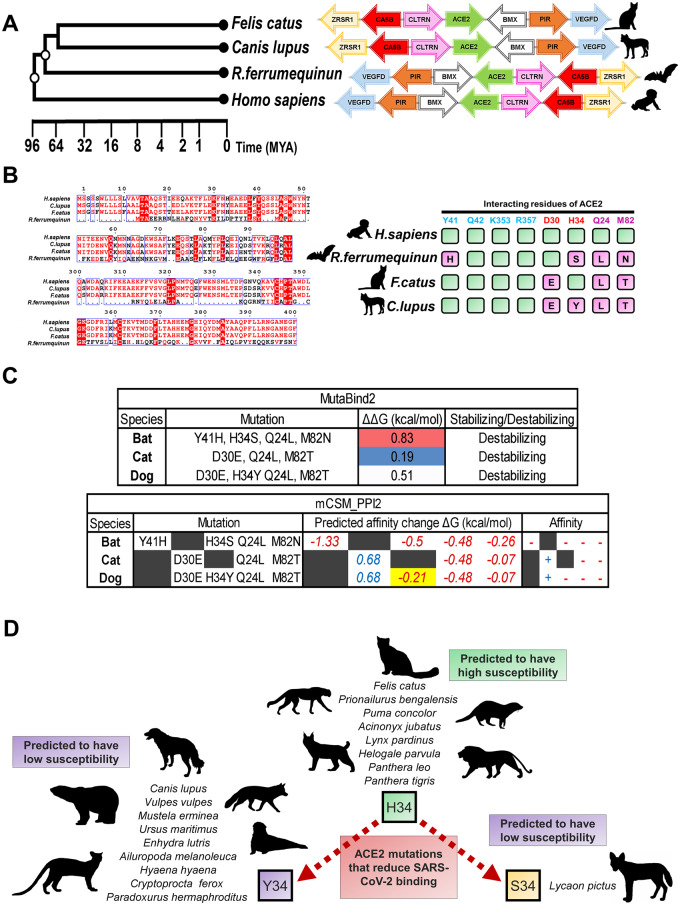
(A) The gene locus of *ACE2* is highly conserved across vertebrates. Phylogeny of cats (*F.catus*), dogs (*C.lupus*), greater horseshoe bat (*Rhinolophus ferrumequinum*) and humans. Tree and estimated times of divergence (MYA, millions of years ago) were obtained from TimeTree (http://www.timetree.org). For each described species, the *ACE2* ortholog gene loci are indicated and conserved genes within the syntenic region are color coded. UCSC Genome Browser (https://genome.ucsc.edu) was used to view the respective of ACE2 loci of each species. Arrows indicate the direction of transcription predicted by UCSC Genome Browser annotations. (B) Alignment of ACE2 protein and its orthologs reveals differences in key residues targeted by SARS-CoV-2. Protein sequences of *F.catus*, *C.lupus*, *R.ferrumequinum* ACE2 were aligned using MUSCLE alignment on the MEGA7 tool (https://www.megasoftware.net/) and sequences from positions 0 to 100 and 300 to 400 are shown. Display for the alignment was generated using the ESPRIPT 3.0 tool (http://espript.ibcp.fr/). Interaction residues between ACE2 and the SARS-CoV-2 spike protein (PDB: 6M17) identified by Yan *et al*.[2] are mapped to the ACE2 orthologs and compared to the human ACE2 green=conserved, magenta=mutated and the letter indicates the residue present in the ortholog. (C) H34Y is predicted to reduce the binding affinity between ACE2 and SARS-CoV-2. MutaBind2 (https://lilab.jysw.suda.edu.cn/research/mutabind2/) and mCSM-PPI2 (http://biosig.unimelb.edu.au/mcsm_ppi2/) analyses reveal changes from mutations in binding affinity between the ACE2 receptor and RBD of the SARS-CoV-2 spike protein. Analyses described in the main text of the article. Affinity indicates the change associated with each binding energy ‘+’ being stabilizing and ‘−’ being destabilizing. (D) H34 mutations in Carnivora predict the susceptibility of species. We analyzed ACE2 orthologs based on sequences from available genomes from Feliforma and Caniforma suborders to determine which species have H34Y mutations. These species with H34S and H34Y mutations are predicted to have reduced susceptibility to the SARS-CoV-2 virus. Silhouettes of organisms from each representative species were obtained from PhyloPic (http://phylopic.org)

A study led by Bu Zhigao and colleagues examined the ability of Severe Acute Respiratory Syndrome coronavirus 2 (SARS-CoV-2), which is responsible for the respiratory syndrome known as COVID-19 (coronavirus disease 2019), to infect and replicate within companion and domesticated animals [1]. One of the companion animals examined were cats (*Felis catus*). Direct exposure to the SARS-CoV-2 virus via the nasal cavity lead to replication of infectious virus in the respiratory tract of domestic cats. This was also the case for ferrets, an animal known to be susceptible to coronaviruses and used for vaccine research, but not dogs (*Canis lupus*), pigs, chickens and ducks. Although some dogs did seroconvert showing antibody responses to the virus, it was surprising that they did not develop active infection since dogs and cats are related carnivores. Utilizing the recently reported crystal structure between angiotensin converting enzyme 2 (ACE2) and the receptor-binding domain (RBD) of the SARS-CoV-2 spike protein [2], we attempted to develop a better understanding of why dogs are less susceptible to SARS-CoV-2 than cats (Fig. 1).

During SARS-CoV-2 infection, the virus targets cell-surface ACE2 through the RBD of the spike protein. Yan *et al*. [2] identified the key interacting residues of ACE2 within this RBD, which includes: Q24, D30, H34, Y41, Q42, M82, K353 and R357. A recent study also identified natural ACE2 missense variants in different human populations [3]. These variants did not overlap with the key residues for interaction with the SARS-CoV-2 spike protein, suggesting that lack of genetic variation in this critical region of ACE2 may have contributed to the rapid global spread of COVID-19. In our analysis, we first examined the syntenic region containing the *ACE2* gene locus in domestic cat and dog, as well as the greater horseshoe bat (a known reservoir for SARS) [4] (Fig. 1A). The goal of these analyses was to better understand how the molecular evolution of ACE2 has contributed to the high susceptibility of some carnivores and low susceptibility of others. As such, our analyses do not address the origins of SARS-CoV-2, which is still a hotly debated topic.

The region of synteny surrounding the *ACE2* gene is highly conserved, allowing us to identify *ACE2* orthologs in each representative species (Fig. 1A). We then aligned the ACE2 proteins encoded by humans, greater horseshoe bat, dog and cat (MUSCLE method) (Fig. 1B). Greater horseshoe bats are natural reservoirs for SARS-like viruses and have adapted to these viruses in part due to changes at key amino acids in their ACE2 receptor [4]. This comparison allowed us to identify changes in the interacting residues that overlap among the four species. Of the spike protein interacting residues, Q24, D30, H34, Y41 and M82 of human ACE2 differ among the species (Fig. 1B). The greater horseshoe bats show variability at 4 of 8 examined residues. Intriguingly, dogs have also a similar number of mutations as the greater horseshoe bat (at differing positions). The key difference between companion animals was a mutation at amino acid H34 only found in dog (H34Y) and not in feline ACE2. Therefore, H34 appears to be a critical residue correlating with susceptibility of species to the SARS-CoV-2 virus.

To model how the species-specific mutations influence the SARS-CoV-2 interaction with the receptor, we performed computation analyses on the human ACE2-RBD SARS-CoV-2 crystal structure (PDB:6M17) using MutaBind2 (Fig. 1C) [5]. These analyses compare binding affinities after mutations to predict whether they stabilize or destabilize the protein–protein interaction by determining the overall change in binding free energies (ΔΔ*G*). The ΔΔ*G* values refer to the change in binding affinity relative to the human ACE2 receptor, based on the amino acid variations observed across the ACE2 receptors from different species. As suspected for a reservoir species that has adapted against the virus, our results predict the greatest ΔΔ*G* and destabilization for the greater horseshoe bat ACE2 and least destabilization from mutations in the cat ACE2 (Fig. 1C). Whereas, dog ACE2 had ∼2.5 times more of a destabilizing ΔΔ*G* in comparison to cats from the H34Y mutation. Similar results were also obtained using mCSM-PPI2 to analyze binding free energy (Fig. 1C) [6]. Together these analyses predict that H34Y would reduce binding affinity between dog ACE2 (vs cat) and the RBD of the SARS-CoV-2 spike protein. As such, our computational analyses support the hypothesis that this single residue difference at H34 in ACE2 could destabilize binding to SARS-CoV-2 and inhibit human–dog transmission of this coronavirus.

We then expanded our analysis and looked at the variability of ACE2 H34 throughout Carnivora, which includes the two suborders of Caniforma and Feliforma. Other species belonging to Canidae (*Vulpes vulpes* and *Lycaon pictus*) and Felidae families (*Prionailurus bengalensis*, *Acinonyx jubatus*, *Puma concolor*, *Lynx pardinus*, *Panthera leo* and *Panthera tigris*) show similar mutations, where H34 is mutated to Y34 (or S34 in the African wild dog *L.pictus*) throughout Canidae and conserved in Felidae (Fig. 1D). In addition, while the H34 mutation was found in all of the Caniforma genomes examined (including Ursidae, Mustelidade and Ailuridae families), that was not the case for Feliforma. It appears that species of Felidae and *Helogale parvula* (dwarf mongoose) are the only Feliforma that are likely susceptible to the virus based on our genome survey. The predicted high susceptibility of Felidae species includes species that are considered endangered or vulnerable, and the virus has the potential to cause devastation in their populations. Indeed, there is a recent report indicating that a tiger at the Bronx Zoo in New York had developed COVID-19 [7]. Sequencing of this virus revealed that it was a human strain of SARS-CoV-2 that infected the tiger. In terms of conservation, it is important to stress that such human-to-cat transmission puts several members of the *Felidae* family in public zoos at risk for COVID-19.

Four interacting residues of ACE2 (amino acids 27, 31, 34 and 82) are noted to influence species specificity of receptor usage by the related SARS-CoV-1 [8]. Furthermore, despite the highly conserved amino acid sequence of ACE2, even single amino acid substitutions such as H34Y found in Canidae have significant consequences on ACE2 binding to the RBD of the SARS-CoV-1 spike protein. These results agree with recent preprint articles that describe the H34Y mutation as reoccurring across different vertebrates (including primates); a mutation suggested to interfere and disrupt the hydrogen bond with Y453 of the spike protein RBD, leading to reduced virus susceptibility [9, 10]. As a consequence, such lock-and-key protein interactions are a limiting step to cross-species susceptibility and zoonotic transmission of the virus. Together this analysis provides a potential mechanistic reason for why dogs and cats differ in susceptibility to SARS-CoV-2.

In different bat species, residues aligning to H34, Q24 and M82 in human ACE2 were identified as being under positive selection among ACE2 receptor homologs [8]. The coevolution between the virus and bat ACE2 receptors is likely the reason why the greater horseshoe was predicted to have low susceptibility to the human strain of the SARS-CoV-2 virus. However, this does not appear to be the case for all horseshoe bats. A related bat species (*Rhinolophus sinicus*) was recently shown to be susceptible to SARS-CoV-2 using an *in vitro* model [11]. Unlike the greater horseshoe bat however, *R.sinicus* ACE2 has mutations in 3 of 8 examined residues, similar to felines [8]. These findings suggest that although horseshoe bats (Rhinolophidae) are reservoirs of the virus, the extent of susceptibility varies between different species. Monitoring of these bat species and determining their susceptibility will be important moving forward to determine which viral reservoirs have the most potential for cross-species transmission.

The positive selection experienced by bats appears to be an evolutionary footprint left by related SARS-like viruses that have driven the coevolution between ACE2 in bats and the coronavirus spike proteins. Similarly, rapid evolution of the ACE2 receptor could have occurred in dog species driven by past canine coronavirus infections. Of note, *ACE2* was previously identified as one of the genes which showed low diversity across the genomes of various domesticated cat breeds [12]. The lack of *ACE2* variation indicates that cats of different breeds may similarly be susceptible. The susceptibility to SARS-CoV-2 is predicted to extend to other members of the *Felidae* family sharing highly homologous ACE2 proteins (Fig. 1B). Although a recent preprint indicated that mammalian species are the most susceptible to the virus, there are also a few fish, bird and reptile species that are likely susceptible [13]. In addition, it was also suggested that rabbits, another common pet, may be more susceptible than cats to the virus [9]. Taken together, the anthroponotic transmission of SARS-CoV-2 to domestic pets including cats has potentially important implications for public health policy regarding cats as a potential vector and reservoir for the SARS-family coronaviruses.

In the greater context of understanding the risks of companion pet zoonosis, there are several examples of pathogens restricted to either cats or dogs based on only a few amino acid differences in viral receptors such as Feline and Canine Parvovirus [14]. As such, evolutionary studies of host receptors have provided powerful rationale for observed differences in viral susceptibility. Although our results provide a possible mechanistic basis for the finding that cats are more susceptibility to SARS-CoV-2 than dogs, more work is still required to better understand the zoonotic potentially of coronaviruses carried by companion animals, and their role in the transmission chain during outbreak tracking. Nonetheless, given the potential for transmission between our feline friends and humans, caution is likely warranted regarding interaction between cats and those most susceptible to COVID-19, including the elderly. Furthermore, such analyses may also aid in identifying the true species-of-origin of SARS-CoV-2, which continues to be debated. Ultimately, the intent of this correspondence is to highlight the power of evolutionary molecular biology to quickly identify biochemically testable hypotheses regarding host–pathogen interactions and the potential for zoonotic transmission.
